# 

**Published:** 1893-07

**Authors:** 


### Notices.

Chicago, July 7, 1893.

From letters received from dentists in different parts of the country, I am inclined to think that there is a lack of information as to the expense of living in Chicago during the season of the World's Fair. Board and lodging were never more reasonable than now. Rooms can be had, where two are willing to room together, for fifty cents a day each. First-class rooms and accommodations can be had from $1.00 to $1.50 per day, when parties wish to room alone. The highest-priced hotels are entertaining people for $5.00 per day, room and board. For twenty cents, in restaurants just outside of the Fair Grounds, will be furnished three eggs, a cup of tea or coffee, and all the bread and butter one wants. For thirty-five cents can be had a good, well-cooked steak, potatoes, tea or coffee, and bread and butter.

The boat, railway and street-car companies are doing all in their power to furnish the best and cheapest transportation possible.

Of course, there are a few catch-penny schemes, but they are not in connection with the responsible hotels or the Fair. Considering the size of the Exposition, their number is remarkably small.

I have made this investigation for the purpose of informing the Dental Profession of the exact facts concerning the expense here, and would urge upon every one to remain as long in Chicago as possible. A month or six weeks can be spent very profitably in seeing what the world has done and is doing, and a longer time could be used to great advantage. This can be done by stopping at a moderate-priced hotel.

The information I have given in this brief article may not be necessary to every one, but I am quite sure that many have been misinformed as to the expense.

Yours cordially, J. N. Crouse.

---

Chicago, July 7, 1893.

For the accommodation of the different Dental Associations which are to meet in Chicago before the convening of the World's Columbian Dental Congress, I have secured the Kindergarten College Hall, 10 Van Buren street, which can be used for all meetings desiring rooms.

For any further information, address

J. N. Crouse, *Chairman.*

---

### American Dental Association.

The Thirty-third Annual Session of the American Dental Association will be held in Kindergarten College Hall, 10 Van Buren street, Chicago, commencing Saturday, August 12th, 1893, at 10 o'clock A. M. Geo. H. Cushing,

*Recording Secretary.*

---

### The National Association of Dental Faculties—Correction.

The next meeting of this body will be held, beginning August 10th, at 10 A. M., in the Kindergarten College Hall, 10 Van Buren street, Chicago, instead of the World's Congress Art Palace, as was heretofore announced. Let all interested give special attention to this change.

A MONTHLY MAGAZINE DEVOTED TO THE INTERESTS OF THE

**DENTAL PROFESSION.**

*Terms Reduced to* $2.00 *Per Year. Single Copies,* 25 *Cents.*

**EDITED BY PROF. J. TAFT, D.D.S.**

—AND—

**Published by SAM'L A. CROCKER & CO., Ohio Dental and Surgical Depot.**

The volume commences in January and closes in December.
The REGISTER being issued monthly is always fresh, therefore Indispensable to the practitioner who desires to keep posted up with the new inventions and improvements which are daily developed. Neither expense nor effort will be spared to give value and variety to its contents. Articles will be illustrated with well executed engravings whenever they will add to the interest or assist to explanation.
Its extensive circulation and frequency of issue make it one of the BEST DENTAL ADVERTISING MEDIUMS in the United States.
All subscriptions must begin with January or July.
Local or special notices, five lines or less, will be inserted for $3 each insertion; over five lines, 50 cts. per line.
All advertising bills payable in advance, or at furthest, after the issue of the first number containing the advertisement. All transient advertisements to be paid for in advance.
☞CONTRIBUTIONS SOLICITED.
Exclusively Editorial Communications should be addressed to the Editor.
The latest number of the REGISTER may be ordered with or without other good' at any time, and all letters should be addressed to

